# Increased Eating Frequency Is Associated with Lower Obesity Risk, But Higher Energy Intake in Adults: A Meta-Analysis

**DOI:** 10.3390/ijerph13060603

**Published:** 2016-06-17

**Authors:** Yue-Qiao Wang, Yun-Quan Zhang, Fei Zhang, Yi-Wen Zhang, Rui Li, Guo-Xun Chen

**Affiliations:** 1 Department of Nutrition and Food Hygiene, School of Public Health, Wuhan University, 185 East Lake Road, Wuhan 430071, China; elle529@126.com (Y.-Q.W.); zhangfei@whu.edu.cn (F.Z.); yiwen315@126.com (Y.-W.Z.); 2 Department of Epidemiology and Biostatistics, School of Public Health, Wuhan University, 185 East Lake Road, Wuhan 430071, China; Yun-quanZhang@whu.edu.cn; 3 Department of Nutrition, University of Tennessee at Knoxville, 1215 West Cumberland Avenue, Knoxville, TN 37996, USA; gchen6@utk.edu

**Keywords:** eating frequency, obesity risk, energy intake, meta-analysis

## Abstract

Body weight is regulated by energy intake which occurs several times a day in humans. In this meta-analysis, we evaluated whether eating frequency (EF) is associated with obesity risk and energy intake in adults without any dietary restriction. Experimental and observational studies published before July 2015 were selected through English-language literature searches in several databases. These studies reported the association between EF and obesity risk (odd ratios, ORs) in adults who were not in dietary restriction. R software was used to perform statistical analyses. Ten cross-sectional studies, consisting of 65,742 participants, were included in this analysis. ORs were considered as effect size for the analysis about the effect of EF on obesity risk. Results showed that the increase of EF was associated with 0.83 time lower odds of obesity (*i.e.*, OR = 0.83, 95% confidence intervals (CI) 0.70–0.99, *p* = 0.040). Analysis about the effect of EF on differences in participants’ energy intake revealed that increased EF was associated with higher energy intake (β = 125.36, 95% CI 21.76–228.97, *p* = 0.017). We conclude that increased EF may lead to lower obesity risk but higher energy intake. Clinical trials are warranted to confirm these results and to assess the clinical practice applicability.

## 1. Introduction

Prevalence of overweight and obesity, which are established risk factors for non-communicable diseases such as cardiovascular diseases, diabetes, and certain cancers, has dramatically increased around the world since 1980. In 2014, 39% and 13% of adults (above 20 years old) were overweight and obese worldwide, respectively [1]. Fortunately, evidence shows that obesity can be prevented by changing potential causes such as dietary behavior and physical activity [2].

The fundamental cause of obesity is an energy imbalance between energy intake (EI) and energy expenditure (EE). Weight gain indicates that the balance has tipped towards increased EI or reduced EE or a combination of both [3]. In humans, when EI exceeds EE by 11 kcal per day, a one pound weight gain will occur over the course of a year [4]. It has been indicated that ingestive behaviors such as choice of foods, time to consume a meal, as well as meal size and function of gastrointestinal system influence EI [5]. For example, EE can drop when there is an increase of sedentary behavior and elevated use of vehicles, which are associated with a decrease of physical activity. Recently, there have been a number of studies investigating the effect of changing dietary behaviors on EI, which is one of goals of the present study.

One of the modifiable dietary behaviors is eating frequency (EF). Commonly, an eating occasion is defined as any instance in which participants reported consumption of solid meals and snacks, with a minimum gap of 15 min between two eating episodes [6]. However, definition of eating occasion can be different, depending on different research. Currently, the relationship between EF and EI is controversial. Animal experiments found that increased EF was associated with higher EI in mice [7], which was in line with observation in human [8]. However, Speechly *et al.* reported that increased EF improved appetite control and subsequently reduced EI in obese males [9]. Interestingly, it has been suggested that increased EF had minimal effect on appetite and EI but reduced EF showed negative impact on appetite and EI based on controlled feeding studies in humans [10].

Despite conclusion about the association between EF and EI is far from consistent, it is plausible that EF may influence at least one side of energy balance and thus affects body weight. Previous evidence from animal studies suggested that the effect of EF on energy balance and body composition was not obvious in rats during caloric restriction [11]. This provided implications for the research on the relationship between EF and body weight without food restriction. Verbaeys *et al.* found that without caloric restriction on a daily basis, rats fed *ad libitum* gained weight faster than three-time schedule-fed rats [12]. For humans, the mainstream opinion is that increased EF is associated with a healthier body weight status in both children and adults [13,14,15]. Since Fabry *et al.* pointed out this inverse relationship in the 1960s [16], numerous cross-sectional studies had been carried out and similar results had been obtained. Recently, Kaisari *et al.* have shown that increased EF was associated with lower obesity risk in children and adolescents, especially in boys [17]. Similarly, Schoenfeld *et al.* suggested a potential benefit of higher EF for body weight status during weight loss, although the positive findings were produced by a single study [18]. However, several studies demonstrated null [19,20] or positive relationship [21,22,23] between EF and body weight. Duval *et al.* suggested that after correcting the effect of physical activity EE, the association between EF and obesity was no longer significant [24]. The present study aims to evaluate whether EF is associated with obesity risk and EI in adults without caloric restriction by conducting a meta-analysis of published original observational studies.

## 2. Methods

This meta-analysis was carried out in accordance with the Preferred Reporting Items for Systematic Reviews and Meta-Analyses (PRISMA) statement [25].

### 2.1. Search Strategy

Original research and observational studies (including those identified via review articles) published before July 2015, examining the association between EF and obesity in adults who were not during weight loss, were selected through English-language literature searches in the PubMed, Elsevier Science Direct, Nature, Science Online and Embase databases. Combinations of at least two of the following key words were used as search terms: meal frequency, feeding frequency, EF, eating patterns, eating behaviors, body composition, weight, body mass index (BMI), obesity and EI. In addition, the reference lists of the eligible retrieved articles were used to identify relevant articles that were not extracted through the searching procedure. Abstracts from conferences, reviews, and unpublished dissertations or theses were excluded from analysis. To reduce the potential for selection bias, each study was independently evaluated by 2 of the investigators (Yue-Qiao Wang and Yi-Wen Zhang), and a mutual decision was made jointly as to whether or not it met the basic inclusion criteria. The disagreements were solved by the opinion of the third author (Fei Zhang), and consensus was reached by discussion. 

### 2.2. Inclusion Criteria

Studies were included if they met the following criteria: (1) original research or observational studies published in an English-language refereed journal; (2) all of the selected studies were conducted in adults; (3) participants or subjects were not during weight loss and diet control; (4) participants or subjects did not have psychological and eating disorders; (5) no experience of bariatric surgery; (6) non-pregnancy; (7) the independent variable was EF; (8) the dependent variable was risk of obesity or EI; (9) results were provided in a form that could be used for the present analysis (point estimates of odd ratios (OR) with 95% confidence intervals (CI) or the differences in the mean values of the EI among EF categories and standard errors (SEs) available).

### 2.3. Data Extraction

The following information was extracted from each included study: first author’s last name; year of publication; country of origin; sample size; age and gender of participants, and the number of daily meals/eating episodes; effect size measurements; 95% CIs; SEs or SDs; the evaluation of EF and EI; the deﬁnition of a meal/eating episode and variables that entered into the multivariable model as potential confounding factors. Information and data was extracted by one author (Yue-Qiao Wang) and checked by another (Yun-Quan Zhang). Quality of studies was not assessed because the total number of studies was limited. 

### 2.4. Quality Assessment

Individual study quality was assessed using a score modified from the Agency for Healthcare Research and Quality (AHRQ) Cross-Sectional Study Quality Assessment [26]. For each study, the appropriateness in dealing with eleven items was checked as follows: (1) define the source of information; (2) list inclusion and exclusion criteria for exposed and unexposed subjects or refer to previous publications; (3) indicate time period used for identifying patients; (4) indicate whether or not subjects were consecutive if not population-based; (5) indicate if evaluators of subjective components of study were masked to other aspects of the status of participants; (6) describe any assessments undertaken for quality assurance purposes; (7) explain any patient exclusions from analysis; (8) describe how confounding was assessed and/or controlled; (9) if applicable, explain how missing data were handled in the analysis; (10) summarize patient response rates and completeness of data collection; (11) clarify what follow-up, if any, was expected and the percentage of patients for which incomplete data or follow-up was obtained. For item 5, a score of 1 was assigned when answer was no, and for other items, a score of 1 was assigned when answer was yes.

### 2.5. Statistical Analysis

For studies investigating the relationship between EF and obesity risk, the effect size was presented as ORs and their corresponding 95% CIs. The lowest category was considered as reference group. If the original study used the highest category as reference, reciprocal of original ORs was calculated for analysis. Single comparison between one EF category *versus* the reference constituted the units of the meta-analysis. When more than one comparisons of different EF categories were included in the same study, an overall estimate for the study was calculated from ORs using the fixed-effect model [27]. For example, if the EF categories included ≤3; 4–5 and ≥6 and EF ≤ 3 was considered as reference group, we used fixed-effect model to combine ORs of other two groups as overall estimate.

For studies investigating the relationship between EF and EI, the effect size was shown as the differences of EI among categories and their corresponding SEs. If the study reported EI in kilojoule or megajoule, we unified the unit as Kcal. In each study, we calculated the differences of EI among categories. For example, if the EF categories included ≤3; 4–5 and ≥6, we calculated the differences of EI between each two groups as well as their corresponding SEs, and computed the overall estimate from the difference of EI using the fixed-effect model. If SEs or SDs were not reported, SEs were computed from 95% CI following a standard methodology [27].

If a paper reported the results of different multivariate models, the most stringently controlled estimate were extracted. Statistical heterogeneity that was attributed to studies rather than to chance was assessed by visual inspection of forest plots as well as using Cochran’s *Q* and *I*^2^, and evaluated by performing the χ^2^ test (assessing the *p* value) [28]. If the *p* value was less than 0.10 and *I*^2^ exceeded 50%, indicating the presence of heterogeneity, a random-effects model (the DerSimonian and Laird method) was used [29]; otherwise, the fixed-effects model (the Mantel–Haenszel method) was used [30]. The presence of publication bias was carried out by visual inspection of asymmetric plots, and tested with the Egger linear regression method of asymmetry [31,32,33]. All statistical calculations were performed in R software (R Foundation for Statistical Computing, Vienna, Austria).

## 3. Results

### 3.1. Study Selection

**Figure 1:**
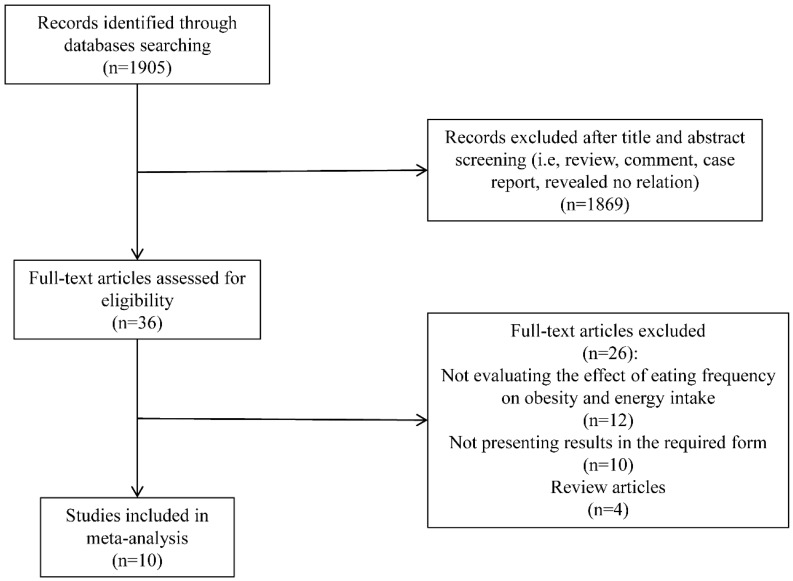
Flow diagram of literature search. Results from the literature search and study-selection procedure are summarized.

Figure 1 shows the results from the literature search and study-selection procedure. A total of 1905 studies were evaluated based on the search criteria. Ten cross-sectional studies that investigated the relationship between EF and obesity risk were identified according to the criteria. No new eligible study was yielded after a manual search of references cited in these articles. Among these 10 studies, five investigated the relationship between EF and obesity risk, and their results were presented as ORs and 95% CIs. Two studies separated results by gender [34,35] and one of these two [34] also separated results by overweight and obesity. Thus, a total of nine sub-studies were included while OR was considered as effect size. In addition, seven out of 10 selected studies investigated the relationship between EF and EI. The results were shown as the differences in the mean values of EI among EF categories.

### 3.2. Study Characteristics

Detailed characteristics of selected studies were presented in Table 1, and results of data extraction were shown in Table 2 and Table 3. For analysis of the association between EF and obesity risk, two studies took the highest EF category as reference group, thus we presented the reciprocal of reported ORs as original ORs in the present study. Also, these two studies had more than one comparisons, we therefore computed combined ORs using the fixed-effect model. [34,35]. For analysis of the association between EF and EI, the unit of EI in three studies was transformed to Kcal [34,36,37] and SE of one study was calculated from 95% CI [38]. All included studies had more than one comparison and we used fixed-effect model to perform the analyses. The overall working sample consisted of 65,742 participants aging from 20 to 89 years old. Ethnically, the subjects in the majority of these studies were Caucasian. No race-related difference was reported in two studies [39,40] involving different races. With regard to the geographical distribution, four studies included Mediterranean populations (Spain, Greece, and Sweden), three included American populations, two included West European populations (France and UK) and one was a cross-continent study (UK and USA). All included studies’ quality score was above 10.

In order to evaluate participants’ dietary behavior, five studies collected data by self-reported questionnaires [34,35,36,41,42], three by food records [37,38,39], and two by 24-h dietary recalls [6,40]. Participants’ weight and height were measured by trained technicians in all selected studies except the one conducted by Mills *et al.* [39]. To compute total EI, published food frequency questionnaires with approved validity and reproducibility were used. Additionally, physical activity level was considered as a potential confounder in the analyses of the relationship between EF and body composition [24]. In most studies, physical activity level was estimated as adjusted parameter. As mentioned in the Methods section, definitions of eating occasion among these cases differed. Three studies estimated EF by defining an eating occasion as any instance in which participants reported consumption of solid meals and snacks, with a minimum gap of 15 min between two eating episodes [6,38,40]. According to Ruidavets *et al.*, however, the minimum gap between two eating episodes was an hour [37]. In four other studies, both meals and snacks were included to evaluate EF [34,36,39,42]; whereas Edelstein *et al.* [41] and Marín-Guerrero *et al.* [35] excluded snacks from their analyses. Four studies excluded drinks from their analyses [6,34,41,42] while others considered that drinks could contribute to EI [35,36,37,38,39,40].

### 3.3. EF and Obesity Risk in Adults

**Figure 2:**
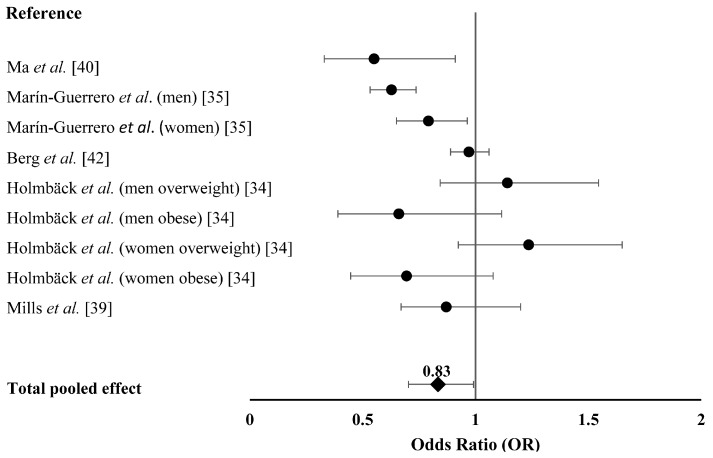
The effect of EF on obesity risk in adults. Forest plot of studies that evaluated the effect of EF on obesity risk in adults (squares and diamonds represent effect size; extended lines show 95% CIs). Increased EF, as compared with the reference category, was inversely associated with obesity risk. EF, eating frequency.

**Figure 3:**
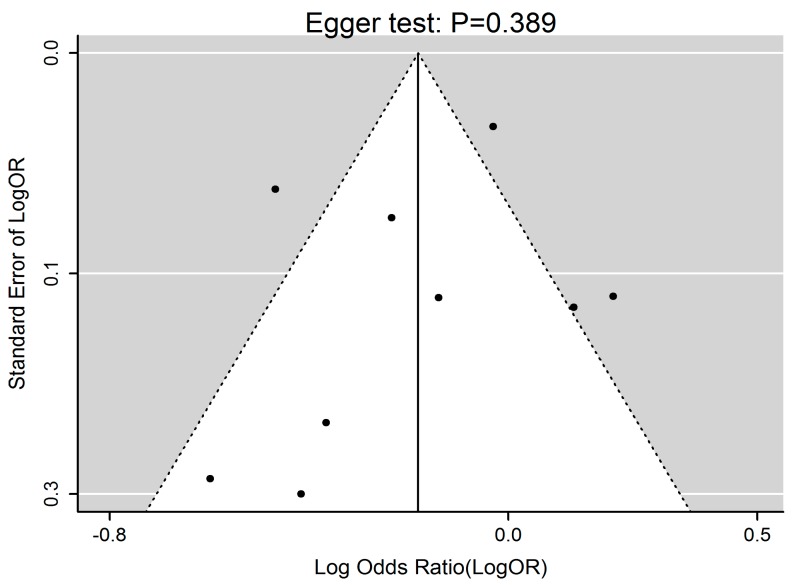
Funnel plot of EF and obesity risk. Funnel plot for 9 studies indicated potential presence of publication bias, while the Egger test revealed no publication bias (*p* = 0.389). EF, eating frequency.

Figure 2 summarized the effect of EF on obesity in adults when considering ORs as effect size. Generally, increased EF was associated with 0.83 time lower risk of obesity (*i.e.*, OR = 0.83, 95% CI 0.70–0.99, *p* = 0.040). A significant heterogeneity of the effect size of EF on obesity was revealed (*p* < 0.001, *Q* = 36.34, *I*^2^ = 77.98%). Publication bias test by visual inspection of asymmetric plot indicated potential presence of publication bias, while the Egger test revealed no publication bias (*p* = 0.389, Figure 3).

### 3.4. EF and Energy Intake in Adults

**Figure 4:**
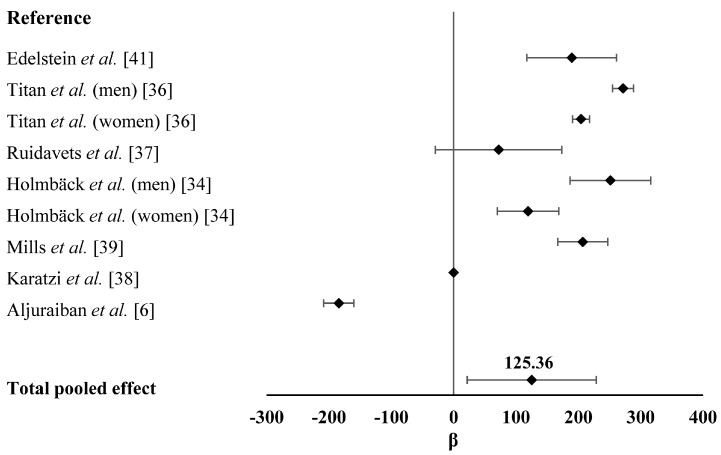
The effect of EF on EI in adults. Forest plot of studies that evaluated the effect of EF on EI in adults (squares and diamonds represent effect size; extended lines show SEs). Increased EF, as compared with the reference category, was positively associated with EI. EF, eating frequency; EI, energy intake.

**Figure 5:**
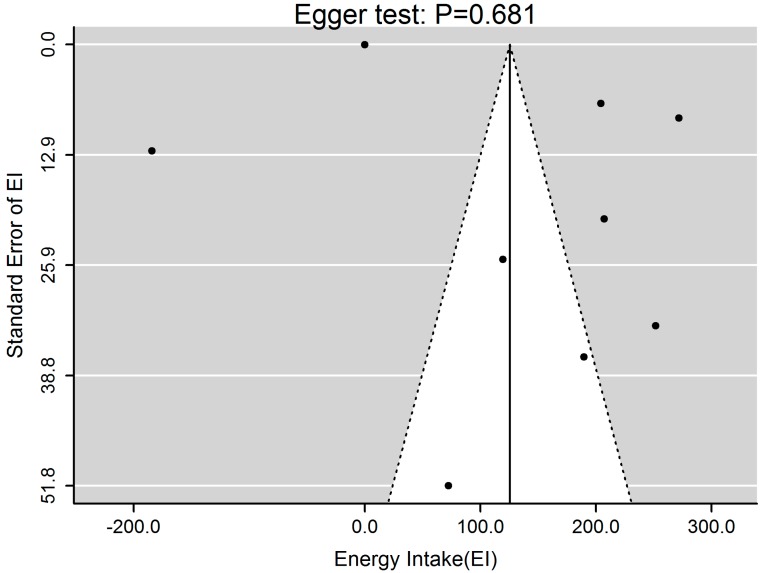
Funnel plot of EF and EI. Funnel plot for 9 studies indicated potential presence of publication bias, while the Egger test revealed no publication bias (*p* = 0.681). EF, eating frequency; EI, energy intake.

Analysis of the effect of EF on differences in participants’ EI was shown in Figure 4. We found that increased EF was associated with higher EI (β = 125.36, 95%CI 21.76–228.97, *p* = 0.017) with significant heterogeneity (*p* < 0.001, *Q* = 106.00, *I*^2^ = 91.51%), which was mainly caused by the studies conducted by Karatzi *et al.* [38] and Aljuraiban *et al.* [6]. They suggested an adverse correlation between EF and EI. Publication bias test by visual inspection of asymmetric plot indicated potential presence of publication bias, while the Egger test revealed no publication bias (*p* = 0.681, Figure 5).

### 3.5. Sensitivity Analysis and Subgroup Analysis

A sensitivity analysis was carried out for each model by conducting a stepwise exclusion of the results of each study. Data were not influenced excessively by omitting any single study, with the values of ORs ranging from 0.795 to 0.892 and βs of EI from 106.29 to 164.99.

Significant heterogeneity introduced a warning about the generalization of the results. Thus, we performed subgroup analyses in order to find out probable causes for the significant heterogeneity. For EF and obesity risk, we found that the results of each subgroup were robust (all ORs <1). All selected studies were adjusted for physical activity. As shown in Table 4, subgroup analysis performed on EF definition (both meals and snacks were included to evaluate EF) had an *I*^2^ value of 41.76%, suggesting that EF definition might be the cause of the significant heterogeneity. In addition, subgroup analyses performed on age (>40 years) and social economic status (adjusted) had lower *I*^2^ values of 53.39% and 53.39%, respectively, meaning that age and social economic status were likely to cause the heterogeneity.

As for EF and EI, we found that *I*^2^ of each subgroup was higher than 50%. Therefore, it was difficult to identify the causes for the significant heterogeneity, which was possibly due to the different study designs among selected articles (Table 5).

## 4. Discussion

To the best of our knowledge, this is the first meta-analysis to assess the effect of EF on obesity risk and EI in adults without caloric restriction. We found a significant and inverse relationship between EF and obesity risk. Adults with high EF had 17% lower probabilities of getting overweight and obese. Our analysis also indicated a significant and positive association between EF and EI in adults.

Several theories have been proposed to explain the negative relationship between EF and obesity risk in adults. For instance, it has been reported that increased EF improves dietary quality and is associated with lower dietary energy density [6] and therefore lower BMI [43,44,45]. Dietary quality refers to the nutrition component of a diet and is measured by evaluating dietary patterns based on national dietary guidelines [46,47]. According to the Healthy Eating Index (HEI) that is developed to evaluate overall diet quality, high dietary quality requires 30% or less EI from fat, less than 10% EI from saturated fat and low protein intake [48]. High EF is usually associated with high EI from carbohydrates but low EI from protein and fat due to the increased consumption of healthy snacks like fruits [34,35,49]. Additionally, high EF may significantly increase the thermic effect of food, which is an important component of EE. Time of eating might be another cause of the negative association between EF and obesity. High EF is usually associated with more eating occasions occurring early in a day, which is more likely to promote EE. Therefore, higher EF may lead to lower obesity risk.

Increased EF may influence hormone secretion and nutrient metabolism, which play important roles in obesity. It has been suggested that increased EF is associated with a reduction in total insulin secretion and better blood glucose control [50]. Jenkins *et al.* found that during a high EF diet, the mean serum insulin level was decreased by approximately 28% [51]. Karatzi *et al.* recruited 164 healthy subjects, measured their plasma levels of insulin and glucose, and found that EF was inversely correlated with insulin concentration and postprandial glucose [38]. Additionally, increased EF may lead to lower serum lipid concentrations [34,51], lower concentrations of total cholesterol and low-density-lipoprotein cholesterol [6,41,52]. These evidence suggest that high EF has a positive effect on nutrient metabolism, which directly contribute to obesity risk.

Several epidemiological studies have suggested that the negative relationship between EF and obesity risk is attributed to reporting bias [20,53], considering that obese and elder individuals may under-report food consumption [14,54]. However, the same association was observed from researches excluding under-reporters [14,34].

It is worth noting that Drummond *et al.* reported this inverse relationship between EF and obesity in males, but not in females [14]. Similar results were obtained from a meta-analysis of the relationship between EF and obesity in children [17], indicating that gender difference should be considered as a cofounding factor in the analysis. However, due to the limited studies in this area, we were unable to analyze the differential effects of EF on obesity risk in males and females.

Our results support a positive relationship between EF and EI. One plausible explanation for the positive association between EF and EI is that higher EF may increase the probability of excessive energy consumption [39]. However, it has been reported that high EF promotes appetite control, and this may result in reduced EI [9,55,56]. Additionally, a recent study has shown that high EF has little influence on appetite control based on an 8-week intervention trial [57]. We speculated that another factor, portion size, might contribute to the reported differential relationships between EF and EI [58]. The portion size of foods, which is commercially available at supermarkets, family-type restaurants and fast food establishments, has been identified as an important factor to affect EI [59,60]. However, despite the effects of portion size should not be neglected, mechanistic evidence suggested that compensatory dietary response to increased EF was weaker than to larger portion size, making high EF a more sensitive factor for EI [61].

It is of interest to find that increased EF is associated with greater EI but lower obesity risk. Previous studies have reported that individuals with higher EF are likely to live a more active lifestyle, leading to higher EI and EE [34,38]. In addition, Duval *et al.* reported that physical activity EE was positively correlated with EF by using more objective measures [24]. This finding may partially explain the different effects of EF on obesity risk and EI. Regarding this discrepancy, an alternative explanation may be that errors commonly attributed to dietary assessment might have occurred during the assessment of these two outcomes. Additionally, difference in dietary quality may also play a role. For example, subjects involved in the studies with higher EF and EI may be eating a diet with relative low caloric content. Nevertheless, the underlying mechanism merits further investigation.

This meta-analysis has several potential limitations. First, only observational studies were included in the analysis and therefore casual inference was limited. For instance, increased EF could be both a cause and consequence of obesity, since obese individuals might increase EF to control body weight. Second, we could only identify cross-sectional studies based on our search strategy because very few studies investigated the associations between EF and obesity risk and/or EI in randomized clinical trials (RCTs). We realized that the evidence level of cross-sectional studies was not as convinced as RCTs and cohort studies; however, considering the current research in EF, it is difficult to perform a meta-analysis on RCTs or cohort studies due to the lack of those studies at the time of our analysis. Third, although we performed subgroup analyses, we were unable to assess the exact effect of every exposure on the observed relationship due to limited selected studies. Third, inconsistent definition for EF should be taken into account. Also, the validity and reliability of food frequency questionnaire and dietary recall to evaluate EF and EI need to be considered. However, this is a common limitation in reviews and meta-analysis evaluating dietary behaviors [62], since there is no general agreement on the appropriate definition and assessment strategy [63]. Finally, misinformation of EI and physical activity was not excluded in several selected studies.

## 5. Conclusions

Results of the present meta-analysis suggest that increased EF is associated with lower obesity risk but higher EI. Despite the aforementioned limitations, these results suggest that increasing EF may benefit body weight management. However, other cofounding factors need to be considered as well, such as portion size. Thus, rigorously designed trials are warranted to confirm these results and to assess the practical applicability.

## Figures and Tables

**Table 1:** Basic characteristics of evaluated studies.

Study [Ref.]	Year	Country	Sample, *n*			EF (No. of Times per Day)	Adjustment Factors	Findings
			Male	Female	Age			
Edelstein *et al.* [41]	1992	USA	2034		50–89	1–2; 3; ≥4	2	BMI did not differ significantly by EF.
Titan *et al.* [36]	2001	UK	6890	7776	45–75	1–2; 3; 4; 5; ≥6	1,5–12	BMI was negatively associated with EF in men but not in women.
Ruidavets *et al.* [37]	2002	France	242	0	45–64	1–2; 3; 4; ≥5	1,4	Increase of EF is associated with lower body fatness.
Ma *et al.* [40]	2003	USA	251	248	20–70	≤3; ≥4	1,2,4,7,13	A greater number of eating episodes each day was associated with a lower risk of obesity.
Marín-Guerrero *et al*. [35]	2008	Spain	16,929	18,045	25–64	1; 2; 3–4	1,3,6,7,9,14,15,20	Obesity was more prevalent in those having only two meals per day than in those having three or four meals in men and women.
Berg *et al.* [42]	2009	Sweden	3591		25–77	1–8	1,2,6,7	There was no significant association between obesity and EF due to methodological problems.
Holmbäck *et al*. [34]	2010	Sweden	892	1024	45–72	≤3; 4–5; ≥6	1,4,6,7,9,11,13,16,17	Men with a low EF showed an increased risk of general and central obesity and results for women showed similar but non-significant tendencies.
Mills *et al.* [39]	2011	USA	0	1099	40–60	1–3; 4; 5; 6; ≥7	1,3,4,13,16,18–21	EF was not associated with overweight/obesity, but it was associated with energy intake.
Karatzi *et al.* [38]	2014	Greece	62	102	46.8 ± 9.3	continuous	1,11–13,22,23	EF was inversely associated with BMI.
Aljuraiban *et al*. [6]	2015	USA & UK	1232	1153	40–59	＜4; 4–5; 5–6; ≥6	1,2,6,7,13,24,25	A larger number of small meals may be associated with improved diet quality and lower BMI.

Adjustment factors: 1: age; 2: gender; 3: race/ethnicity; 4: education; 5: obesity; 6: cigarette smoking; 7: physical activity; 8: intake of calories; 9: alcohol; 10: protein; 11: fat; 12: carbohydrate; 13: total energy intake; 14: health status; 15: lifestlye; 16: socio-economic status; 17: fibre intake; 18: other eating behaviors; 19: MET min/week; 20: marital status; 21: menopausal status; 22: HDL; 23: HOMA-IR; 24: dietary; 25: population sample. Ref.: reference; EF: eating frequency; BMI: body mass index.

**Table 2:** ORs and 95% CIs between EF and obesity.

Study [Ref.]	Year	EF	Original OR	Combined OR	95% CI
Ma *et al.* [40]	2003	continuous	0.55	0.55	0.33–0.91
Marín-Guerrero *et al.* [35] (Men)	2008	3 or 4	0.7	0.63	0.47–1.06
		2	0.61		
Marín-Guerrero *et al.* [35] (Women)	2008	3 or 4	0.9	0.79	0.56–1.41
		2	0.77		
Berg *et al.* [42]	2009	continuous	0.97	0.97	0.89–1.06
Holmbäck *et al.* [34] (Men overweight)	2010	≥6	1.06	1.14	0.62–1.85
		4~5	1.17		
Holmbäck *et al.* [34] (Men obese)	2010	≥6	0.41	0.66	0.17–0.98
		4~5	0.87		
Holmbäck *et al.* [34] (Women overweight)	2010	≥6	1.32	1.24	0.64–2.70
		4~5	1.22		
Holmbäck *et al.* [34] (Women obese)	2010	≥6	0.39	0.69	0.14–1.08
		4~5	0.79		
Mills *et al.* [39]	2011	continuous	0.87	0.87	0.49–1.30

Original OR: original OR was directly extracted from included studies using the lowest EF category as reference. If the original study used the highest EF category as reference group, we presented the reciprocal of reported ORs as the Original OR in the table. Ref.: reference; EF, eating frequency.

**Table 3:** βs and *SE*s (standard errors) for the differences in EI among EF categories.

Study [Ref.]	Year	EF	EI (kcal)	β	SE
Edelstein *et al.* [41]	1992	1–2	1962 ± 45.4	189.46	36.65
		3	1792 ± 26.2		
		4	1658 ± 63		
Titan *et al.* [36] (Men)	2001	1–2	1965.8 ± 621.9	271.82	8.61
		3	2027 ± 552.5		
		4	2232.8 ± 616.4		
		5	2383.4 ± 641.5		
		≥6	2542.8 ± 692.9		
Titan *et al.* [36] (Women)	2001	1–2	1810.9 ± 571.2	204.25	6.92
		3	1786.3 ± 482.1		
		4	1925 ± 528.2		
		5	2054 ± 531.1		
		≥6	2213.7 ± 602.3		
Ruidavets *et al.* [37]	2002	1–2	2306.4 ± 645.3	72.23	51.75
		3	2380.5 ± 597.5		
		4	2414 ± 645.3		
		≥5	2485.7 ± 621.4		
Holmbäck *et al.* [34] (Men)	2010	≤3	2557.4	251.52	32.99
		4–5	2724.7		
		≥6	2963.7		
Holmbäck *et al.* [34] (Women)	2010	≤3	1959.8	119.50	25.19
		4–5	2103.3		
		≥6	2175		
Mills *et al.* [39]	2011	≤3	1864 ± 583	207.01	20.47
		4	2025 ± 627		
		5	2158 ± 765		
		6	2235 ± 630		
		≥7	2348 ± 730		
Karatzi *et al.* [38]	2014	continuous	0.03	0.03	0.01
Aljuraiban *et al.* [6]	2015	≤4	2472	−184.23	12.49
		4–5	2402		
		5–6	2294		
		≥6	2129		

EI, energy intake (kcal): transformed data were presented; Ref.: reference; EF, eating frequency.

**Table 4:** Subgroup analysis for EF and obesity risk.

Exposure	Subgroup	Number of Studies	*Q*	*p*-Value	*I*^2^ (%)	OR (95% CI)
Gender	male	3	11.75	0.003	82.99%	0.78 (0.51,1.20)
	female	4	7.48	0.058	59.92%	0.89 (0.71,1.12)
	mixed	2	4.66	0.031	78.58%	0.77 (0.45,1.33)
Age	>20	4	25.56	<0.0001	88.27%	0.75 (0.57,0.97)
	>40	5	8.58	0.072	53.39%	0.94 (0.74,1.18)
Country	USA	2	2.36	0.124	57.65%	0.73 (0.47,1.13)
	Med	7	32.98	<0.0001	81.81%	0.86 (0.71,1.05)
Education	unadjusted	3	22.53	<0.0001	91.12%	0.79 (0.59,1.04)
	adjusted	6	13.08	0.023	61.77%	0.87 (0.68,1.11)
Smoking	unadjusted	2	2.36	0.124	57.65%	0.73 (0.47,1.13)
	adjusted	7	32.98	<0.0001	81.81%	0.86 (0.71,1.05)
Alcohol	unadjusted	3	5.02	0.081	60.20%	0.85 (0.67,1.09)
	adjusted	6	23.34	0.0003	78.58%	0.83 (0.65,1.07)
SES	unadjusted	4	25.57	<0.0001	88.27%	0.75 (0.57,0.97)
	adjusted	5	8.58	0.072	53.39%	0.94 (0.74,1.18)
Fiber Intake	unadjusted	5	25.57	<0.0001	84.36%	0.77 (0.62,0.96)
	adjusted	4	7.77	0.051	61.40%	0.95 (0.70,1.28)
EF definition	1	6	8.59	0.13	41.76%	0.96 (0.83,1.11)
	2	2	3.22	0.0726	68.97%	0.7 (0.56,0.88)
	3	1	0	1		0.55 (0.33,0.91)
Diet Assessment	self-report	7	32.98	<0.0001	81.81%	0.86 (0.71,1.13)
	food records	1	0	1		0.87 (0.65,1.16)
	dietary recalls	1	0	1		0.55 (0.33,0.91)
Reference Group	EF = 1	2	3.22	0.0726	68.97%	0.7 (0.56,0.88)
	EF = 3	4	7.77	0.051	61.4%	0.95 (0.7,1.28)
	EF was continuous	3	5.03	0.0811	60.2%	0.85 (0.67,1.09)
Total		9	36.34	<0.0001	77.98%	0.83(0.70,0.99)

SES: social economic status; Med: Mediterranean; EF (eating frequency) definition: 1: both meals and snacks were included to evaluate EF; 2: excluded snacks to evaluate EF; 3: an eating occasion as any instance in which participants reported consumption of solid meals and snacks, with a minimum gap of 15 min between two eating episodes.

**Table 5:** Subgroup analysis for EF and EI.

Exposure	Subgroup	Number of Studies	*Q*	*p*-Value	*I*^2^ (%)	β (95% CI)
Gender	male	3	14.66	0.001	86.36%	211.64 (121.28,301.99)
	female	3	10.69	0.005	81.29%	181.47 (136.54,226.39)
	mixed	3	244.50	<0.0001	99.18%	−2.39 (−151.72,146.94)
Education	unadjusted	5	2112.69	<0.0001	99.81%	95.52 (−44.97,236.01)
	adjusted	4	16.54	0.001	81.86%	168.69 (99.90,237.48)
Obesity	unadjusted	7	429.35	<0.0001	98.60%	91.44 (−7.71,190.59)
	adjusted	2	37.43	<0.0001	97.33%	237.84 (171.63,304.05)
Smoking	unadjusted	4	130.94	<0.0001	97.71%	116.86 (−19.31,253.03)
	adjusted	5	978.60	<0.0001	99.59%	132.10 (−32.41,296.61)
Alcohol	unadjusted	5	348.72	<0.0001	98.85%	54.18 (−64.21,172.57)
	adjusted	4	56.66	<0.0001	94.71%	213.13 (158.69,267.56)
PA	unadjusted	3	28.67	<0.0001	93.02%	84.44 (−45.45,214.33)
	adjusted	6	982.47	<0.0001	99.49%	144.53 (1.66,287.41)
SES	unadjusted	5	2114.63	<0.0001	99.76%	91.91 (−36.71,220.54)
	adjusted	3	11.96	0.003	83.28%	190.89 (119.52,262.27)
EF definition	1	5	51.52	<0.0001	93.05%	212.11 (166.56,257.66)
	2	2	217.64	<0.0001	99.54%	−91.68 (−272.25,88.89)
	3	1	0	1		189.46 (117.63,261.29)
	4	1	0	1		72.23 (−29.2,173.66)
Diet Assessment	self-report	5	57.67		93.06%	209.42 (160.63,258.21)
	food records	3	104.19		98.08%	93.13 (−64.79,251.05)
	dietary recalls	1	0	1		−184.23 (−208.71,−159.75)
Reference Group	EF = 1–2	3	38.67	<0.0001	94.87%	226.28 (170.14,282.43)
	EF = 3	3	13.10	0.0014	84.74%	151.58 (49.92,253.23)
	EF = 4	2	266.31	<0.0001	99.62%	11.06 (−372.35,394.46)
	EF was continuous	1	0	1		
Total		9	2297.53	<0.0001	99.65%	125.36(21.76,228.97)

PA, physical activity; SES, social economic status; EF (eating frequency) definition: 1: both meals and snacks were included to evaluate EF; 2: an eating occasion as any instance in which participants reported consumption of solid meals and snacks, with a minimum gap of 15 min between two eating episodes; 3: excluded snacks to evaluate EF; 4: the minimum gap between two eating episodes was an hour.

## Abbreviations

The following abbreviations are used in this manuscript:

EF	eating frequency
EI	energy intake
EE	energy expenditure
BMI	body mass index
SES	social economic status
Med	Mediterranean
PA	physical activity
OR	odd ratios
CIs	confidence intervals
SEs	standard errors
